# Substitution in Prescriptions

**Published:** 1896-08

**Authors:** 


### SUBSTITUTION IN PRESCRIPTIONS.

The complaint that druggists substitute one preparation or drug for another ordered by the physician is constantly reappearing. The reader can learn more of this by reading the article reproduced in "Medical Items Continued," p. x. No matter what the doctor orders, it is the business of the druggist to fill the prescription as written, unless it contains an error dangerous to life. Druggists show many favors to doctors, but some of them often take liberties with prescriptions.

